# The Use of Herbal Medicines Among Cancer Patients

**DOI:** 10.7759/cureus.53455

**Published:** 2024-02-02

**Authors:** Ana Carolina Vasques, Patricia Cavaco, Tânia Duarte, Vanessa Duarte Branco, Mafalda Miranda Baleiras, Marta Pinto, Filipa Ferreira, Maria de Fátima Falcão, Tiago Dias Domingues, Ana Martins

**Affiliations:** 1 Medical Oncology, Centro Hospitalar Lisboa Ocidental, Lisbon, PRT; 2 Hospital Pharmacy, Centro Hospitalar Lisboa Ocidental, Lisbon, PRT; 3 Statistics and Operations Research, Faculty of Sciences of the University of Lisbon, Lisbon, PRT

**Keywords:** pharmacokinetics, adverse effects, cancer patients, interactions, herbal medicine

## Abstract

Background and objective

The use of herbal medicines has been increasing among cancer patients, as a way to control cancer and treatment-related symptoms; however, many patients are reluctant to disclose this use to their medical practitioners. The fact that oncological treatments have a narrow therapeutic margin, associated with the lack of control and clinical evidence concerning these supplements, makes medication-herbal interactions a reality. These interactions could lead to increased toxicity or a decreased effectiveness of oncological treatment. In light of this, we aimed to assess the prevalence of herbal medicine use in a patient population at a Portuguese central hospital: Centro Hospitalar Lisboa Ocidental.

Materials and methods

Patients with breast, prostate, or colorectal cancer diagnoses between August 2022 and July 2023 and undergoing oncological treatment were included. Data were collected through a survey during their first appointment, as well as by consulting the patients' clinical files. An interaction evaluation was carried out to assess potential medication-herbal interactions. Finally, a statistical analysis was performed to identify predictive factors for the use of herbal medicines.

Results

Among the 65 patients included in the study, 52% were females, and the median age of the cohort was 65 years. Breast cancer was the most prevalent diagnosis and the majority of the patients were undergoing palliative treatment. We found that 46% of patients used herbal medicines regularly: to strengthen the immune system, detoxification of the body, and treat insomnia and constipation. A medication-herbal interaction was found in 37% of the cases, the most frequent being doxorubicin-vitamin C, through an antioxidant mechanism. The univariable analysis failed to show any predictive factors associated with the use of herbal medicines.

Conclusions

This study sheds light on herbal medicine use among cancer patients and the reality of medication-herbal interactions. There is an urgent need for further research and evidence-based medical protocols regarding herbal medicine use, especially in complex cases such as cancer patients, to provide better and safer care.

## Introduction

It is estimated that between 15 and 73% of cancer patients in Europe use herbal medicines to complement anticancer treatments or minimize their adverse effects [1-8]. Up to 70% of patients tend to not spontaneously disclose it to their oncologist or pharmacist, as they are perceived to be natural, safe, or devoid of any harmful potential [1,3,6,8-9]. However, oncological treatments are highly complex and involve various types of drugs, often in combination, which have a great potential for drug interactions [1,3,7]. There are risks associated with the concomitant use of herbal medicines because they use the same metabolic and transport pathways as anticancer treatments, which can result in adverse reactions and drug interactions [1,3-6,9]. Adverse reactions can manifest varying levels of severity, ranging from allergic reactions to gastrointestinal problems or even serious cases of hepatotoxicity, neurotoxicity, or nephrotoxicity.

A drug interaction is defined as a pharmacological response to the administration or co-exposure of a drug with another substance that modifies the patient's response to that drug, which may trigger serious adverse effects, such as changes in the drug's efficacy or toxicity [6-7,9]. The best-known metabolic and transport pathways associated with pharmacokinetic interactions of clinical relevance are hepatic cytochrome P450 isoenzymes, glucuronidation enzymes, and renal or intestinal transmembrane transporters (PgP, MRP1-2, AOT, OCT). Interactions are especially significant in cancer patients, given that antineoplastics have a narrow therapeutic margin with inherent toxicity, even at therapeutic concentrations [1,9]. In addition, there are major discrepancies in the definition and categorization of herbal medicines, which are purchased without a prescription and sold without any rigorous controls or regulations over their quality, safety, and effectiveness [1,5-7,9]. Many of these products constitute mixtures of herbs that do not adhere to any regulation for their cultivation, extraction, and preparation [5,9]. Also, the lack of randomized clinical trials affects the ability to evaluate the efficacy and safety of these products alone and in association with oncological treatment, which also raises concerns about their use [1-7,9-11].

Several factors have been positively correlated with herbal medicine’s use, such as young age, high education level, high income, female gender, marital status, and uncontrolled symptoms. Therefore, it is important to characterize the use of these products in cancer patients, to acquire greater knowledge about the potential benefits and risks associated with them, including possible harmful interactions [1,3-4,6,8]. The main objective of this study is to characterize the use of herbal medicines among cancer patients, particularly those with breast, prostate, and colorectal cancer, which have the highest incidence rates. Newly diagnosed cancer patients at a central hospital were asked to fill out a questionnaire at the first medical appointment. Our secondary objective involves evaluating the possible pharmacokinetic and pharmacodynamic interactions of herbal medicines with the prescribed antineoplastics.

## Materials and methods

We employed a descriptive, observational, and prospective design to conduct this study. Data were collected through a survey among patients, along with consulting their clinical files. We obtained informed consent from patients to access their electronic files. The survey was filled out with the support of one researcher, who clarified any doubts. This study was approved by the institutional ethics committee (approval number: 2189) and has been carried out by adhering to the tenets of the Declaration of Helsinki.

Interaction analysis was carried out by using the Lexicomp® Drug Interactions databases, the Memorial Sloan Kettering Cancer Center “About Herbs, Botanicals & Other Products” search engine, and the Stockley’s Herbal Medicines Interactions (1st Edition, 2009) manual, published by Pharmaceutical Press. In the event of any potentially serious interaction, the patient was informed about the same for their benefit.

The inclusion criteria were as follows: patients diagnosed with breast, prostate, or colorectal cancer, between August 2022 and July 2023, at all stages of the disease and undergoing oncological treatment. Patients who were not eligible for oncological treatment and who only had indications for symptom control were excluded from the study. The patients who used herbal medicines were subjected to a further sub-analysis, which involved assessing their gender, age, education, location of the primary neoplasm, and disease stage, intending to determine and characterize the features of this patient population.

Statistical analysis

Quantitative variables were presented as mean ± standard deviation (SD), while qualitative variables were documented as frequencies and percentages. To identify the predictive factors for the use of herbal medicines, multivariable binary logistic regression was constructed by considering the use/nonuse of herbal medicines as the dependent variable. Statistical analysis was performed using the R software version 4.2.2. A p-value less than 0.05 was considered statistically significant.

## Results

This study lasted 12 months (August 2022-July 2023), and a total of 65 patients were included. Of these, 52% were female (n=34) and the median age of the sample was 65 years (SD: 14 years). Breast cancer was the most common diagnosis (n=32, 49%), followed by prostate cancer (n=17, 26%) and colorectal cancer (n=16, 25%) (Table 1). Most of the patients (n=28, 43%) were in stage IV and were undergoing palliative treatment, while 23 received adjuvant treatment (35%), and 14 neoadjuvant treatments (22%). Regarding education, most of the patients had completed high school, followed by those with education up to 4th (n=15, 23%) and 9th grade (n=15, 23%), while 14 patients (22%) had university-level education. A significant majority of the patients (82%, n=53) were taking some form of medication for a comorbidity.

**Table 1:** Demographic characteristics of the study population

Variable	N (%)
Gender	
Female	34 (52%)
Male	31 (48%)
Age	
Median	65 ± 14 years
Type of cancer	
Breast	32 (49%)
Prostate	17 (26%)
Colorectal	16 (25%)
Treatment type	
Neoadjuvant	14 (22%)
Adjuvant	23 (35%)
Palliative	28 (43%)
Educational level	
4th grade	15 (23%)
9th grade	15 (23%)
12th grade	21 (32%)
University education	14 (22%)

Regarding the use of herbal medicines, around 30 patients (46%) reported using them, with the stated aim of strengthening the immune system, detoxification of the body, and treating insomnia and constipation. Of these, 70% (n=21) had used it after the cancer diagnosis, and 60% (n=18) used it daily. Regarding the source of information about herbal medicine, family and friends were the most common ones (50%, n=15), and the most common source of supply was a herbalist (57%, n=17). Around half of the herbal medicines did not come with any leaflet explaining their side effects and dosage. Of the patients who did not use any supplement, the majority (57%, n=20) justified this decision by stating that they did not feel the need. Finally, the vast majority of patients who used herbal medicines (77%, n=23) were unaware of the potential harmful effects of their use concomitantly with antineoplastic agents (Table 2). In 60% of appointments (n=39), this subject was not addressed, and the most common reason stated (28%, n=18) was that the health professional had not asked about it.

**Table 2:** Characteristics of study patients who use herbal medicines

Variable	N (%)
Herbal medicine use	
Yes	30 (46%)
No	35 (54%)
Use after diagnosis	
Yes	21 (70%)
No	9 (30%)
Source of supply	
Herbalist	17 (57%)
Pharmacy	6 (20%)
Supermarket	4 (13%)
Other	3 (10%)
Knowledge of interactions	
Yes	7 (23%)
No	23 (77%)
Interactions found	
Yes	11 (37%)
No	19 (63%)

Drug interactions with anticancer agents were found In 37% of cases (n=11) with herbal medicine use; doxorubicin and vitamin C constituted the most common interaction (10%, n=3), followed by doxorubicin and green tea (7%, n=2). The most frequently involved enzyme complexes were CYP3A4, CYP2D6, and P-glycoprotein, and the inhibition mechanism was the most common type of mechanism (Table 3). Based on the univariable analysis, no possible predictive factors were associated with the use of herbal medicines (Table 4).

**Table 3:** Interactions found between herbal products and oncological treatment

Interactions	N (%)	Mechanism
Doxorubicin - vitamin C	3 (10%)	Antioxidant
Doxorubicin - green tea	2 (7%)	Inhibition CYP3A4
Doxorubicin - valerian	1 (3%)	Inhibition CYP3A4; inhibition CYP2D6; inhibition glycoprotein-P
Letrozole - valerian	1 (3%)	Inhibition CYP3A4; inhibition CYP2D6; inhibition glycoprotein-P
Letrozole - red rice	1 (3%)	Inhibition CYP3A4; inhibition CYP2D6; inhibition glycoprotein-P
Paclitaxel - aloe vera	1 (3%)	Inhibition CYP3A4
Paclitaxel - valerian	1 (3%)	Inhibition CYP3A4; inhibition CYP2D6; inhibition glycoprotein-P
Paclitaxel - hibiscus	1 (3%)	Induction glycoprotein-P
Irinotecan - turmeric	1 (3%)	Inhibition CYP3A4
Irinotecan - rhodiola	1 (3%)	Inhibition CYP3A4
Capecitabine - folic acid	1 (3%)	Inhibition CYP3A4

**Table 4:** Univariable binary logistic regression results with the use/non-use of herbal medicines as the dependent variable

Variables	b	SE	OR (95% CI)	P-value
Age (years)	-0.017	0.018	0.98 (0.95-1.02)	0.33
Gender				
Female vs. male	-0.39	0.50	0.68 (0.25-1.82)	0.44
Cancer type				
Colorectal vs. breast	-0.28	0.64	0.76 (0.21-2.60)	0.66
Colorectal vs. prostate	-1.28	0.75	0.28 (0.06-1.16)	0.09
Educational level				
4th vs. 9th grade	0.27	0.74	1.31 (0.31-5.75)	0.71
4th vs. 12th grade	-2e-17	0.69	1.00 (0.25-4.02)	1.0
4th vs. university education	1.32	0.79	3.75 (0.83-19.46)	0.09
Treatment				
Adjuvant vs. neoadjuvant	0.85	0.69	2.34 (0.61-9.78)	0.22
Adjuvant vs. palliative	0.04	0.57	1.04 (0.34-3.23)	0.95
Stage				
I vs. II	1.20	1.01	3.33 (0.49-29.91)	0.23
I vs. III	0.29	1.01	1.33 (0.19-11.91)	0.78
I vs. IV	0.62	0.95	1.86 (0.31-15.07)	0.51

## Discussion

To our knowledge, this is one of the first studies to assess the use of herbal medicines in a Portuguese population of cancer patients, diagnosed with breast, colorectal, and prostate cancers. This assessment was carried out during the first oncology appointment through a survey involving a questionnaire that aimed to evaluate the use of herbal medicines and the patient's knowledge about them. Almost half of the patients who completed the questionnaire (n=30, 46%) admitted using or having used herbal medicines at some point. These results are slightly on the higher side when compared to those reported in some European studies. In the study by Wode et al., published in 2019, the authors reported that 34% of patients used, or were still using, herbal medicines at the time of filling out the questionnaire [11]. This difference may be attributed to the differences in sample size or the selection of oncological malignancies that the questionnaire addressed.

In contrast with previous studies, we could not find any predictive factors for the use of herbal medicines in our sample. However, the literature shows that the use of these types of products was more prevalent among female, younger, and more educated patients [1,3,6,11]. Patients with higher education levels are better informed about other treatment options and are likely to have sufficient resources to afford them [6]. The most frequently mentioned source of information about the use of these products was family and friends. This becomes especially relevant given that this is not information based on scientific evidence. Only four patients reported obtaining information about herbal medicines from a healthcare professional.

Around 70% (n=21) of patients had used herbal medicines after being diagnosed with cancer, the main objectives being to strengthen the immune system, “detoxification of the body”, and treat insomnia and constipation. it appears that these products are seen as something that complements conventional antineoplastic treatment, and not as their replacement, which is also in line with previous studies [2,3,6,11]. Also, patients’ desire to play a more proactive role in their treatment seems to play an important role in their use of herbal medicines [6]. The concomitant use of herbal medicines and anticancer treatments represents an immense challenge in the management of cancer patients, in light of the potential interactions between these products and cancer treatments [1,3,4]. In our study, drug interactions with anticancer agents were observed in 37% of cases (n=11) with herbal medicine use. Herbal products use the same metabolic and transport pathways as onco-directed treatments, which can lead to potential interactions. Cytochrome P450 was most frequently associated with potential drug interactions in this study.

Most patients were unaware that the use of these products could lead to toxicity or drug interactions with oncological treatment. Moreover, the patient was asked about the use of herbal medicines in only 40% of appointments (n=26). This highlights the need to raise awareness among oncologists about the prevalence of herbal medicine use among cancer patients, and it is mandatory to question the patient about their use of these products before starting antineoplastic treatment [3,4,6,11]. It is crucial to gain deeper insights into this topic by conducting randomized clinical trials, to provide better care by avoiding unwanted pharmacological interactions that could compromise the efficacy and safety of oncological treatments [3,4,6,11]. This information may also help guide healthcare and public health policies and encourage patients to seek professional advice regarding herbal medicine use [4,6].

This study has a few limitations, especially related to its short duration and limited sample size; moreover, an element of selection bias could have crept in since we only included patients with breast, prostate, and colorectal cancer from a single institution in an urban area of a developed country. Certain types of cancers and treatments were not represented in our study. Also, due to the recall bias caused by the fact that patients self-reported the use of herbal medicines, the study did not address the experiences of patients who decided not to disclose or did not remember its use.

## Conclusions

Almost half of our sample of cancer patients reported using herbal medicines. Many cancer patients use them regardless of the healthcare professional's opinion about their benefits. Our findings highlight the need to raise awareness among healthcare professionals and patients about the use of these types of products. We recommend conducting similar studies involving this type of questionnaire among other oncological patients to determine the true extent of the use of herbal medicines. We also strongly recommend concomitant randomized clinical trials exploring herbal medicine use among cancer patients.
